# Supplementary material for: The effects of exercise on body composition of prostate cancer patients receiving androgen deprivation therapy: An update systematic review and meta-analysis
Source: PLoS One. 2022 Feb 15;17(2):e0263918. doi: 10.1371/journal.pone.0263918 (PMC8846498; doi:10.1371/journal.pone.0263918)
Supplement: S2 File — (DOCX) [file pone.0263918.s002.docx]

**S2 File. Search strategies for databases.**

**PubMed**

(exercise*[Ti/Ab] OR training*[Ti/Ab] OR physical activity*[Ti/Ab]) AND (prostate cancer [Ti/Ab] OR PCa [Ti/Ab]) AND (Androgen deprivation therapy [Ti/Ab] OR ADT [Ti/Ab])

**Web of Science**

AB= (exercise* OR training* OR physical activit*) AND AB= (prostate cancer OR PCa) AND AB= (Androgen deprivation therapy OR ADT).

**Embase**

(exercise* OR training* OR physical activit*) [Abstract] AND (prostate cancer OR PC*) [Abstract] AND (Androgen deprivation therapy OR ADT) [ Abstract].

**EBSCO**

(exercise* OR training* OR physical activit*) [Ab] AND (prostate cancer OR PCa) [Ab] AND (Androgen deprivation therapy OR ADT) [Ab].

**Cochrane Library**

(exercise* OR training* OR physical activit*) [Ti/Ab/Kw] AND (prostate cancer OR PCa) [Ti/Ab/Kw] AND (Androgen deprivation therapy OR ADT) [Ti/Ab/Kw].
